# Gender, Race/Ethnicity, Interpersonal Discrimination, and Cognitive Performance: An Intersectional Analysis

**DOI:** 10.1093/geroni/igaf122.2460

**Published:** 2025-12-31

**Authors:** Natalie Rivadeneira, Julia Libby, Timothy Hohman, Ganga Bey

**Affiliations:** The University of North Carolina at Chapel Hill, Chapel Hill, North Carolina, United States; Vanderbilt University Medical Center, Nashville, Tennessee, United States; Vanderbilt University Medical Center, Nashville, Tennessee, United States; UNC Chapel Hill, Chapel Hill, North Carolina, United States

## Abstract

Previous research has shown that interpersonal discrimination affects cognitive health disparities across gender and race/ethnicity. However, the impact of belonging to multiple social groups on these experiences is rarely considered. We applied an intersectional perspective to examine how gender, race/ethnicity, and interpersonal discrimination intersect to socially pattern cognitive performance in a cohort of US adults aged 50 and older. Using pooled data from Kaiser Healthy Aging and Diverse Life Experiences (KHANDLE) and Study of Healthy Aging in African Americans (STAR) cohorts (n = 2,622), we applied multilevel analysis of individual heterogeneity and discriminatory accuracy (MAIHDA) to investigate variation in executive function, memory, and language across 24 intersectional strata defined by gender, race/ethnicity, and self-reported experiences of everyday and major lifetime discrimination. Model main effects indicate that the additive effects of gender, race/ethnicity, and discrimination experience explained most between-strata variance for all cognitive domains. Variables that define intersectional strata accounted for 17.9% of overall variance in executive function for strata that included everyday discrimination, and 17.1% of variance for strata that included lifetime discrimination. The effects of stratum-defining variables were modest for memory and language (7.7%-11.6%). However, examining by level of perceived discrimination shows cognitive performance variation within and between ethnoracially and gender-defined groups in all cognitive domains. While the effect of discrimination exposure on cognitive performance varied, the additivity of effects suggests that intervention strategies to promote equity in cognitive health should be universal while tailored to the specific needs of ethnoracially and gender-defined groups experiencing discrimination.

